# Preliminary Evaluation of the African American Alzheimer’s Caregiver Training and Support Project 2 (ACTS2) Facebook Live Workshop Series: A Mixed Methods Analysis

**DOI:** 10.1002/alz.091090

**Published:** 2025-01-09

**Authors:** Nik M. Lampe, Alexandra C. H. Nowakowski, Tomeka Norton‐Brown, Joshua Paredes, Skyler Bastow, Marin Savage‐Paik, Jayson Bakshi, Amar Patel, Robert L. Glueckauf

**Affiliations:** ^1^ University of South Florida, Tampa, FL USA; ^2^ Florida State University College of Medicine, Orlando, FL USA; ^3^ Florida State University College of Medicine, Tallahassee, FL USA; ^4^ Florida State University, Tallahassee, FL USA

## Abstract

**Background:**

The African‐American Alzheimer’s Caregiver Training and Support Project 2 (ACTS2) is a faith‐integrated, skills‐training and support program for Black family caregivers of persons living with dementia in Florida. In response to the COVID‐19 pandemic, ACTS2 initiated a bi‐monthly Facebook Live Workshop series, offering practical information and resources for Black communities on dementia caregiving topics (e.g., caregiver self‐care and negotiating stressful caregiving situations). Using mixed assessment methods, we evaluated viewer reach, engagement, and satisfaction with the workshops.

**Methods:**

We used quantitative (Facebook and YouTube usage statistics, online pre‐registrations, online evaluation surveys) and qualitative data (viewer engagement during workshops, online evaluation surveys) to evaluate viewer reach, engagement, and satisfaction with 22 workshops from bi‐monthly ACTS2 Facebook Live Workshops series, delivered from May 2020 to August 2023.

**Results:**

The ACTS2 Facebook Live Workshop series reached a total of 1,193 individuals who pre‐registered (averaging 52 per workshop) and at least 1,032 individuals who attended the virtual workshops in live time (averaging 45 per workshop). To date, the series reached at least 9,697 Facebook user views and 274 YouTube user views. Thematic analysis of attendee engagement revealed three themes: (1) requesting additional information and community resources; (2) sharing personal information about caregiving experiences; and (3) gratitude towards workshop presenters and presentations. From the pool of 1,193 workshop pre‐registrants, 98 completed evaluation surveys. Nine out of ten survey completers reported the workshops were “extremely informative” or “very informative” (92.9%) and rated the overall quality of workshops as “excellent” or “good” (90.8%). Four themes emerged from the qualitative analysis of survey data: (1) gratitude for workshop presenters’ testimonies; (2) satisfaction with ACTS2 and other community resources; (3) centering Black dementia caregiver experiences; and (4) concerns with attendee/presenter communication format during workshops.

**Conclusion:**

Initial findings of the mixed methods analysis suggested that the ACTS2 Facebook Live Workshop series was successful in reaching and engaging community members across Florida and the U.S., while serving as a useful tool in providing information and community resources for Black dementia caregivers. Further research is needed to enhance online communication between participants and presenters during live workshops and accrual of evaluation surveys.

